# The Effect of Concurrent Auditory Working Memory Task in Auditory Category Learning

**DOI:** 10.3390/bs15040440

**Published:** 2025-03-31

**Authors:** Jie Wu, Jianghong Lu, Zixuan Che, Siying Li

**Affiliations:** 1 Department of Psychology, Fujian Normal University, Fuzhou 350117, China; wuj@fjnu.edu.cn (J.W.); qsx20220443@student.fjnu.edu.cn (J.L.); qsx20230462@student.fjnu.edu.cn (Z.C.); 2 Faculty of Education, Northeast Normal University, Changchun 130024, China

**Keywords:** auditory category learning, auditory working memory, rule-based, information-integration, drift-diffusion model

## Abstract

In the auditory domain, the role of auditory working memory in shaping strategy selection and performance within both auditory rule-based and information-integration tasks remains unclear. To address this issue, the present study utilized a concurrent auditory working memory paradigm to investigate the impact of working memory on rule-based and information-integration category learning within the auditory domain. Additionally, we employed a categorization strategy model and drift-diffusion model to examine the impact of auditory working memory on auditory category learning. The categorization strategies model revealed that significantly more participants employed the optimal strategy in the control condition compared to the concurrent working memory condition in both the rule-based and information-integration tasks. Furthermore, given that most participants used the rule-based strategy in the information-integration task, the results showed a decrease in accuracy and an increase in reaction time for the concurrent working memory condition relative to the control condition in rule-based category learning. According to the drift-diffusion model, this decline observed under the concurrent working memory condition can be attributed to a reduction in information accumulation speed, increased cautious decision-making, and longer nondecision time. This study suggests that the concurrent working memory task interfered with participants’ strategy selection and performance, with decreased performance in the concurrent working memory condition stemming from slower information accumulation and extended nondecision time for the rule-based strategy, while more cautious decision-making was related to the information-integration strategy.

## 1. Introduction

Category learning is a cognitive skill that involves perceiving the overarching framework of encounters and recognizing shared characteristics among specific occurrences, thereby facilitating purposeful classification and conceptualization (Seger & Miller, 2010). Various theories and models have been proposed by researchers to elucidate categorization behavior, such as the competition between verbal and implicit systems (COVIS) theory (Ashby et al., 1998). The COVIS theory proposes that the process of category learning involves distinct categorization systems, namely the explicit categorization system and the procedural categorization system (Ashby et al., 1998).

The COVIS theory is supported by a substantial body of literature comparing performance in rule-based (RB) and information-integration (II) categorization tasks. In the rule-based task, individuals engage in classification based on predefined rules, typically expressed using linguistic terms. This approach proves to be the most effective approach in such tasks. For instance, a rule may state that gratings with frequencies below 300 belong to category A, while those above 300 belong to category B. Rule-based category learning involves a cognitive process wherein individuals establish abstract logical rules through hypothesis testing (Ashby et al., 1998; Ashby & Maddox, 2005, 2011). This rule-based strategy relies on the explicit learning system and is supported by brain regions such as the prefrontal cortex, parietal lobe, medial prefrontal cortex, and thalamus (Carpenter et al., 2016; Koenig et al., 2005; Milton et al., 2009; Nomura et al., 2007). In contrast to rule-based tasks where gratings are defined by a single attribute like duration and frequency alone, information-integration tasks often define them based on both duration and frequency. However, direct linguistic description is insufficient for comparing frequency and duration (Miles & Minda, 2011). The information-integration strategy is a cognitive process whereby individuals establish associations between stimuli and key responses (Ashby et al., 1998; Ashby & Maddox, 2005, 2011), which is mediated by the procedural learning system. For instance, the dopamine reward system significantly contributes to establishing motor associations between stimuli and key responses (Ashby et al., 1998).

Previous studies have predominantly focused on visual category learning, whereas in real-life situations, people obtain information from various senses, including auditory, visual, and olfactory perception. In the auditory domain, some evidence also supports the COVIS theory in auditory category learning (Derawi et al., 2023; Gabay et al., 2023; Maddox et al., 2013, 2014; Roark et al., 2024). For example, Gabay et al. (2023) and Roark et al. (2024) found that individuals with dyslexia, who are linked to a selective disruption in the procedural memory system, exhibit impaired auditory information-integration category learning but spared auditory rule-based category learning. Furthermore, although many previous studies have found that visual rule-based learning has significant advantages (Little et al., 2011; Nosofsky et al., 1994), some studies also suggested that participants preferred to use a rule-based strategy in auditory category learning (Wu et al., 2018, 2020). Additionally, some studies have found that the performance of cross-modal category learning is superior to visual category learning, but there was no significant difference between cross-modal and auditory category learning in the rule-based task (Maddox et al., 2006; Smith et al., 2014). This study also suggests that auditory category learning may have an advantage in the rule-based task. Therefore, it is imperative to first investigate the application of category strategies in auditory category learning.

Furthermore, working memory (WM) is a limited capacity system that temporarily stores and processes information (Baddeley, 1992). Given the separate mediation of rule-based category learning and information-integration category learning by the explicit and procedural learning systems (Ashby et al., 1998; Ashby & Maddox, 2005, 2011), it is expected that working memory may have distinct roles in these two types of tasks. Previous studies have indicated negligible impact of working memory on implicit learning (Barnes et al., 2008; Bo et al., 2011, 2012; Brown et al., 2010; Janacsek & Nemeth, 2013; Kaufman et al., 2010; Nemeth et al., 2012; Sefcsik et al., 2009; Vicari et al., 2007) and attention (Curran & Keele, 1993; Frensch et al., 1998; Jiménez & Vázquez, 2005; Jiménez & Méndez, 2001). Conversely, when the learning process is explicit or intentional, working memory becomes involved in guiding attention focus and cognitive control (Bo et al., 2012; Feldman et al., 1995; Unsworth & Engle, 2005). Many studies investigated whether working memory plays different roles in rule-based and information-integration category learning. Accordingly, some results indicate that the concurrent working memory task is likely to exerts a more substantial influence on explicit category learning, such as the rule-based task, while leaving a relatively minor impact on implicit category learning, such as the information-integration task (Miles & Minda, 2011; Wu & Fu, 2021; Zeithamova & Maddox, 2006, 2007). Furthermore, recent studies found working memory ability is related to the speed or accuracy of category learning (Craig & Lewandowsky, 2012; Lewandowsky et al., 2012; Lloyd et al., 2019; McHaney et al., 2021; Souza et al., 2024). Specifically, some studies have found that working memory is associated with higher accuracy, as well as faster and slightly more cautious decision-making in non-native speech category learning (Souza et al., 2024).

However, these studies failed to investigate which specific aspects of category learning are influenced by working memory, such as whether working memory impacts the selection of learning strategies or whether it influences the decision-making stage or nondecision-making processes, which remains under-explored. This area remains under-explored. It is imperative to further investigate the potential differential effects of working memory on auditory category learning and explore its impact on aspects such as strategy selection, accuracy, rate of information accumulation, nondecision time, or level of caution during decision-making. The emergence of the categorization strategy model (e.g., BIC) and the drift-diffusion model (DDM) has addressed some of these issues (Paulon et al., 2020; Ratcliff, 1978). BIC can determine whether the classification strategy employed by participants is the rule-based strategy, the information-integration strategy, or an alternative approach based on their responses. DDM posits that decision-making is a dynamic process involving the accumulation of evidential information, thereby elucidating it as an ongoing sampling procedure encompassing various cognitive components by incorporating reaction time and accuracy measures (Li et al., 2023). This approach allows for a detailed examination of how working memory tasks influence specific components of the classification process, including the rate of information accumulation, nondecision time, and decision-making threshold.

The present study aimed to investigate the impact of auditory working memory on auditory rule-based and information-integration tasks. To address these issues, we employed a concurrent task experimental paradigm combined with a categorization strategy model (BIC) and drift-diffusion model (DDM). Specifically, we incorporated a concurrent auditory working memory task and utilized BIC and DDM to examine its effects on auditory rule-based and information-integration category learning. Participants were assigned to either the control condition or the concurrent working memory condition. In the control condition, a categorization task was administered to assess rule-based category learning or information-integration category learning. In contrast, in the concurrent working memory condition, participants completed an additional auditory working memory task. It is hypothesized that auditory working memory may have a significant impact on overall performance in category learning.

## 2. Method

### 2.1. Participants

Before running the experiment, G-power was used to estimate the sample size. Referring to previous studies (Wu & Fu, 2021), a 2 × 2 between-subjects designs would require an N of 20 in each condition to achieve a power of 0.85, at alpha = 0.05, with a main or interaction effect size of 0.4. Thus, to be conservative, ninety-four participants were compensated for their involvement in the study. The group consisted of 36 males and 58 females, with an average age of 20.32 ± 2.29 years. Participants were randomly assigned to either the concurrent working memory condition or the control condition. After the initial grouping, in the rule-based task, the concurrent working memory condition comprised a total of 23 individuals, and the control condition consisted of 26 individuals; in the information-integration task, the concurrent working memory condition comprised a total of 23 individuals, and the control condition consisted of 22 individuals. Participants whose mean accuracy or reaction time across all trials fell below two standard deviations were excluded from the analysis. The exclusion of one participant resulted in a final sample size of 22 participants for the concurrent working memory condition in the rule-based task. Similarly, one participant was excluded, resulting in a final sample size of 22 participants for the concurrent working memory condition in the information-integration task. All participants had normal vision and normal hearing, and each participant in the experiment wore headphones. All of them received payment for their participation. All enrolled participants were non-music majors in their current academic program. None of them had previously taken part in similar experiments. All participants provided us with informed consent forms. This research received approval from the Research Ethics Committee of the School of Psychology, Fujian Normal University (No. PSY220032).

### 2.2. Materials

**Figure 1:**
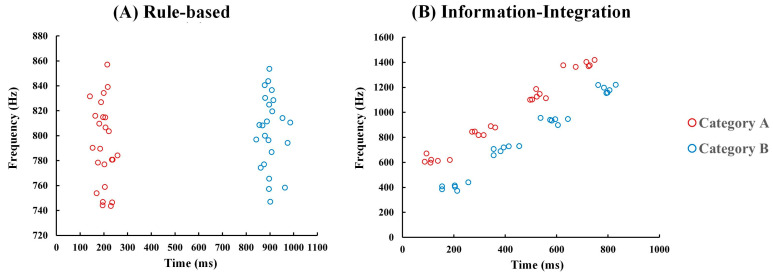
Category structure in both the rule-based task and the information-integration task. (**A**) The category structure in the rule-based task; (**B**) the category structure in the information-integration task.

In the rule-based task, the auditory stimuli were pure tones generated using Adobe Audition 2019. The pure tone was composed of unidimensional structures, encompassing variations in frequency and duration. Specifically, the average value for category A in the time dimension was 200 ms, while for category B, it was 900 ms. In terms of frequency, the mean values were 800 Hz for each respective category. Each stimulus in both categories was generated using a bivariate normal distribution with a standard deviation of 30 and no correlation coefficient (Table 1). According to this methodology, a total of 48 stimuli were randomly generated with an equal distribution of 24 stimuli in each category (see Figure 1. Each stimulus was replicated four times, resulting in a cumulative count of 192 trials. In the information-integration task, the auditory stimuli varied simultaneously in both duration and frequency dimensions. For each category, auditory stimuli were generated using four bivariate normal distributions with a standard deviation of 30 and zero correlation coefficient. Six stimuli were randomly drawn from each distribution, resulting in a total of 24 stimuli per category and 48 stimuli overall. Table 1 presents the corresponding distribution parameters for each category structure. Both sets of distributions for the category structure were created using R 4.2.1 software. All auditory materials were generated using Adobe Audition 2019.

Furthermore, for the concurrent working memory task, the sound stimulus material for the digit sequences was generated using Adobe Audition 2019 on a computer. All voices used were female. The four digits were uniformly recorded with a recording time of 2000 ms set in the program and an equal interval between each digit. In each trial of the experiment, the digits were presented audibly and not visually displayed on the screen.

### 2.3. Procedures

Referring to previous studies (Miles & Minda, 2011; Wu & Fu, 2021; Zeithamova & Maddox, 2006, 2007), in order to investigate how a concurrent working memory task influences categorization, researchers have often conducted a control condition and a concurrent condition. In the control condition, our participants were required to complete a categorization task, while in the concurrent working memory condition, they were instructed to complete both the categorization task and an additional task involving working memory.

**Figure 2:**
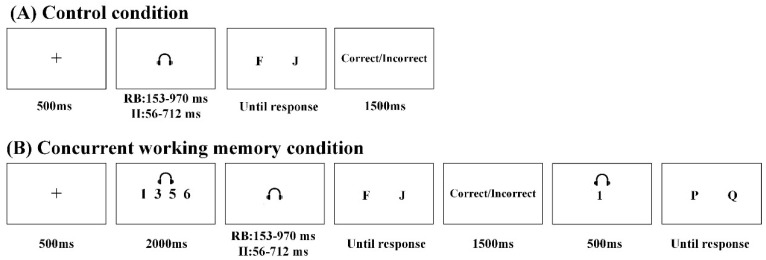
Trial procedure in the rule-based task and the information-integration task. (**A**) The trial procedure in the control condition; (**B**) the trial procedure in the concurrent working memory condition.

In the control condition, participants were instructed to perform a rule-based task or an information-integration task (Figure 2A). Each trial begins with the display of a fixation for a duration of 500 ms, followed by the presentation of auditory stimuli. In the rule-based task, auditory stimuli were presented for durations ranging from 153 ms to 970 ms, whereas in the information-integration task, the auditory stimuli were presented for a duration ranging from 56 ms to 712 ms. Subsequently, a screen displaying either “F” or “J” was presented until a response was made. Participants were then required to indicate whether the presented auditory stimuli belonged to category A or B by pressing either the “F” or “J” keys on their keyboard. The assignment of response keys was balanced across participants. Following each response, correct or incorrect feedback was displayed for 1500 ms. The experiment consisted of four blocks, totaling 192 trials.

In the concurrent working memory condition, participants were instructed to complete both the categorization task and an additional task involving working memory (Figure 2B). Each trial began with a fixation displayed for 500 ms, followed by the presentation of four digits in a vocal sequence lasting for 2000 ms. Participants were explicitly instructed to remember these digits throughout the entire trial. Subsequently, auditory stimuli were presented, and the auditory stimuli in the concurrent working memory condition were identical to those in the control condition. Participants had to classify them into either category A or B by pressing the corresponding keys “F” or “J”. The assignment of response keys was counterbalanced among participants. After each response, feedback indicating correctness or incorrectness was shown for 1500 ms. Then, a single test digit was presented in the auditory modality. Participants needed to indicate whether it belonged to the initial set of four digits they heard at the beginning of the trial by pressing either “P” or “Q”. Half of these test digits had been part of the initial set while half had not. The assignment of response keys was counterbalanced between participants. If a participant made two consecutive errors in judgment, the screen prompt reminding them of their consecutive errors would be triggered.

### 2.4. Data Analysis

#### 2.4.1. Drift-Diffusion Model Analysis

The key parameters involved in the DDM are drift rate ‘*v*’, bias ‘*z*’, boundary height ‘*a*’, and nondecision time ‘*t*’. The ‘*v*’ represents the velocity at which an individual accumulates evidence by continuously gathering information from the starting point to make a decision. The higher the drift rate, the more rapid the accumulation of information. ‘*a*’ reflects the level of information accumulation and the level of caution in decision-making. A larger ‘*a*’ value suggests a stronger focus on accuracy, resulting in more cautious decision-making. ‘*z*’ demonstrates how prior knowledge influences an individual’s judgment; initially assuming equal influence before making a judgment, over time, response preferences toward two options become inconsistent, leading to different values for ‘*z*’. Nondecision time ‘*t*’ refers to processes preceding an individual’s keystroke response, reflecting their readiness to encode information and perform actions (Ratcliff & Mckoon, 2008). In this study, we combined the DDM with the above parameters (i.e., ‘*v*’, ‘*a*’) to analyze and quantify how a concurrent working memory task impacts participants’ speed of information accumulation and the level of caution in decision-making.

The current study utilized the HDDM (Hierarchical Drift-Diffusion Model) toolkit, which is based on the Python 3.10 platform, to analyze the accuracy and reaction time (Wiecki et al., 2013). The dataset utilized for fitting the model consisted of binary responses (correct or incorrect) and their reaction times in concurrent working memory condition and control condition under the rule-based task and the information-integration task. All the trials of each participant under each condition were combined without distinguishing between different blocks. A correct response was denoted as “1” while an incorrect response was denoted as “0”, and data from trials with reaction times of more than 5 s were excluded. By utilizing hierarchical Bayesian parameter estimation in HDDM, it fits parameters at both the individual and population levels, allowing for the examination of differences in estimated posterior parameters to quantify the impact of working memory on rule-based and information-integration category learning within the auditory domain.

#### 2.4.2. Categorization Strategies Analysis

We utilized decision-bound modeling to analyze the strategy types employed by the subjects in the experiment. Given that the experiment was divided into two categories, we fitted four category decision-bound models: an information-integration model, explicit conjunctive and unidimensional models, and a random responder model. The unidimensional model assumes that subjects categorize based on a single dimension, such as categorizing solely according to sound frequency. The explicit conjunctive model posits that subjects combine two dimensions for categorization, for example, integrating sound frequency and duration. The information-integration model assumes that subjects consider the simultaneous change in two dimensions, which can be represented by a diagonal linear decision boundary. The random responder model assumes that subjects’ category judgments are randomized. We fitted these models using the number of experimental trials per subject per block, determined the parameters of each model via the maximum likelihood method, and selected the optimal strategy for subjects’ category judgments based on the Bayesian Information Criterion (BIC). Models were fitted to data from 4 blocks with 192 trials per subject, and the model with the smallest BIC value for each group was chosen as the best-fitting model. Finally, binomial tests were used to compare the proportion of participants in the concurrent working memory and control conditions that best fit the data for each model under the RB and II tasks.

## 3. Results

### 3.1. Categorization Strategies Revealed by the Model Analysis

Table 2 provides an analysis of the subject’s strategies under various conditions, along with the corresponding accuracy rates. In the rule-based task, a significantly higher proportion of participants (0.92) in the control condition utilized unidimensional rule compared to those in the concurrent working memory condition (0.59), *χ*^2^ = 9.93, df = 1, *p* < 0.01, φ = 0.45. This indicates that fewer participants in the concurrent working memory condition relied on unidimensional rules, suggesting that the concurrent working memory task influenced their categorization strategies. In the information-integration task, while a majority of participants in both conditions used unidimensional rules (0.68 in the concurrent working memory condition vs. 0.45 in the control condition), at least 32% of the participants in the control condition employed information-integration strategies, whereas none did so in the concurrent working memory condition. The difference between the two conditions was significant, *χ*^2^ = 8.32, df = 1, *p* < 0.01, φ = 0.43. These findings suggest that the concurrent working memory task had a more pronounced effect on categorization strategies in the information-integration task.

### 3.2. Accuracy

**Figure 3:**
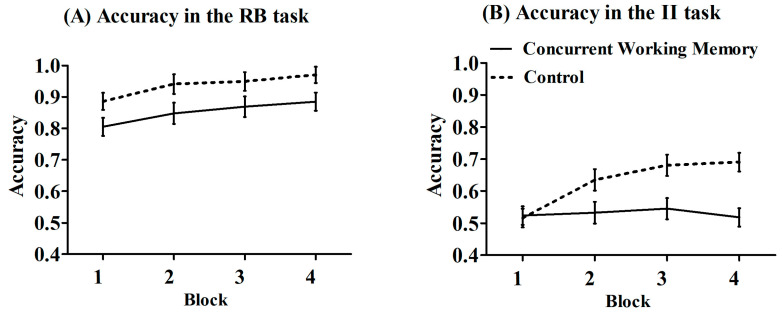
Results for accuracy. (**A**) The accuracy in the rule-based task; (**B**) the accuracy in the information-integration task. Note: The error bar represents the standard error. RB represents rule-based; II represents information-integration.

To examine the potential impact of engaging in a concurrent working memory task on the rule-based task and the information-integration task, a 2 (condition: dual working memory condition vs. control condition) × 2 (task: rule-based vs. information-integration) × 4 (block:1 to 4) mixed-design ANOVA was conducted on accuracy (Figure 3). The main effect of condition was found to be significant, *F* (1, 88) = 12.32, *p* = 0.001, *η_p_*^2^ = 0.12, with significantly higher accuracy observed in the control condition (*M* = 0.78, *SE* = 0.02) compared to the concurrent working memory condition (*M* = 0.69, *SE* = 0.02). Additionally, a significant main effect of task was observed, *F* (1, 88) = 140.52, *p* < 0.001, *η_p_*^2^ = 0.62, indicating that accuracy in the rule-based task (*M* = 0.89, *SE* = 0.02) was significantly higher than in the information-integration task (*M* = 0.58, *SE* = 0.02). Furthermore, a significant main effect of block was identified, *F* (3, 264) = 19.12, *p* < 0.001, *η_p_*^2^ = 0.18. Post hoc analysis revealed that the accuracy of block 1 was significantly lower than blocks 2–4 (*ps* < 0.001), and the accuracy of block 2 was significantly lower than block 3 (*p* = 0.006) and block 4 (*p* = 0.008), but there was no significant difference between block 3 and block 4 (*p* = 0.53). It suggested a learning effect for both tasks. The interaction between block and condition was significant, *F* (3, 264) = 5.15, *p* = 0.002, *η_p_*^2^ = 0.06. Post hoc analysis revealed that the control condition exhibited significantly higher accuracy than the concurrent working memory condition in block 2 (*p* = 0.04), block 3 (*p* = 0.001), and block 4 (*p* < 0.001). The two-way interaction between block and task was not significant, *F* (3, 264) = 0.60, *p* = 0.61, *η_p_*^2^ = 0.007. The two-way interaction between task and condition was not significant, *F* (1, 88) = 0.09, *p* = 0.77, *η_p_*^2^ = 0.001. Notably, the three-way interaction between task, condition, and block was significant, *F* (3, 264) = 4.92, *p* = 0.002, *η_p_*^2^ = 0.05. Post hoc analysis revealed that in the information-integration task, the control condition exhibited significantly higher accuracy than the concurrent working memory condition in block 2 (*p* = 0.03), block 3 (*p* = 0.004), and block 4 (*p* < 0.001). In the rule-based task, the control condition exhibited significantly higher accuracy than the concurrent working memory condition in block 1 (*p* = 0.04), block 2 (*p* = 0.04), and block 4 (*p* = 0.03).

### 3.3. Reaction Time

**Figure 4:**
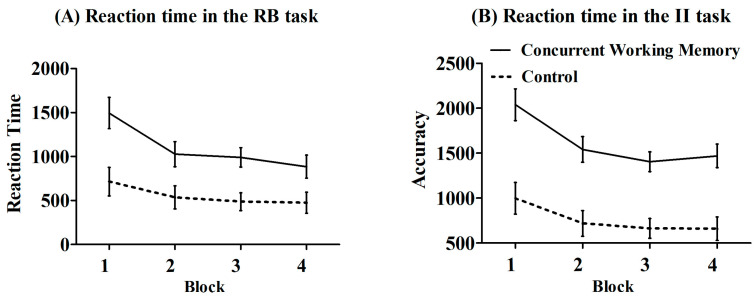
Results for reaction time. (**A**) The reaction time in the rule-based task; (**B**) the reaction time in the information-integration task. Note: The error bar represents the standard error. RB represents rule-based; II represents information-integration.

To investigate the potential impact of a concurrent working memory task on the rule-based task and the information-integration task, a 2 (condition: concurrent working memory condition vs. control condition) × 2 (task: rule-based vs. information-integration) × 4 (block:1 to 4) mixed-design ANOVA was also conducted on reaction time (Figure 4). The results revealed a significant main effect of condition, *F* (1, 88) = 33.98, *p* < 0.001, *η_p_*^2^ = 0.28. Post hoc analysis indicated that participants’ reaction time was significantly slower in the concurrent working memory condition (*M* = 1356.41, *SE* = 82.63) compared to the control condition (*M* = 656.20, *SE* = 83.23), suggesting that engaging in an auditory working memory task could lead to a delayed response time. The main effect of the task was significant, *F* (1, 88) = 9.00, *p* = 0.003, *η_p_*^2^ = 0.09. Post hoc analysis revealed that the reaction time was significantly slower in the information-integration task (*M* = 1186.59, *SE* = 86.63) compared to the rule-based task (*M* = 826.03, *SE* = 83.23). Additionally, there was a significant main effect of block, *F* (3, 264) = 25.39, *p* < 0.001, *η_p_*^2^ = 0.22. The results of post hoc comparisons revealed a significant decrease in reaction time during blocks 2–4 compared to block 1 (*ps* < 0.001), and the reaction time in block 2 was significantly slower than block 3 (*p* = 0.045). The two-way interaction between block and task was not significant, *F* (3, 264) = 0.39, *p* = 0.76, *η_p_*^2^ = 0.004. The two-way interaction between task and condition was not significant, *F* (1, 88) = 1.64, *p* = 0.20, *η_p_*^2^ = 0.02. The two-way interaction between block and condition was significant, *F* (3, 264) = 2.98, *p* = 0.03, *η_p_*^2^ = 0.03. Post hoc analysis revealed that the reaction time was slower for the concurrent working memory condition compared to the control condition across all blocks (*ps* < 0.001). The three-way interaction between block, task, and condition was not significant, *F* (3, 264) = 0.20, *p* = 0.90, *η_p_*^2^ = 0.00.

### 3.4. The Impact of Concurrent Working Memory on Rule-Based Strategy

Considering that most participants employed a rule-based strategy in both the rule-based and information-integration tasks, we aggregated the participants who employed the rule-based strategy from both the experimental and control groups for further analysis. A 2 (condition: concurrent working memory condition vs. control condition) × 4 (block: 1 to 4) mixed-design ANOVA was conducted on accuracy and reaction time. Firstly, for accuracy, the main effect of the condition was significant, *F* (1, 83) = 10.56, *p* = 0.002, *η_p_*^2^ = 0.11. The results suggested that the concurrent working memory task interfered with the rule-based strategy. The main effect of block was significant, *F* (3, 249) = 18.83, *p* < 0.001, *η_p_*^2^ = 0.19, suggesting a learning effect. The interaction was significant, *F* (3, 249) = 18.83, *p* = 0.002, *η_p_*^2^ = 0.06. Post hoc analysis revealed that accuracy in the control condition was significantly higher than in the concurrent working memory condition across blocks 2 to 4 (*ps* < 0.001). Secondly, for reaction time, the main effect of condition was significant, *F* (1, 83) = 29.01, *p* < 0.001, *η_p_*^2^ = 0.26. The results suggested that the concurrent working memory task interfered with the rule-based strategy. The main effect of block was significant, *F* (3, 249) = 22.43, *p* < 0.001, *η_p_*^2^ = 0.21. The interaction was significant *F* (3, 249) = 2.90, *p* = 0.04, *η_p_*^2^ = 0.03. Post hoc analysis revealed that the reaction time was faster in the control condition than in the concurrent working memory condition across all blocks (*ps* < 0.001).

### 3.5. Drift-Diffusion Model Parameters

**Figure 5:**
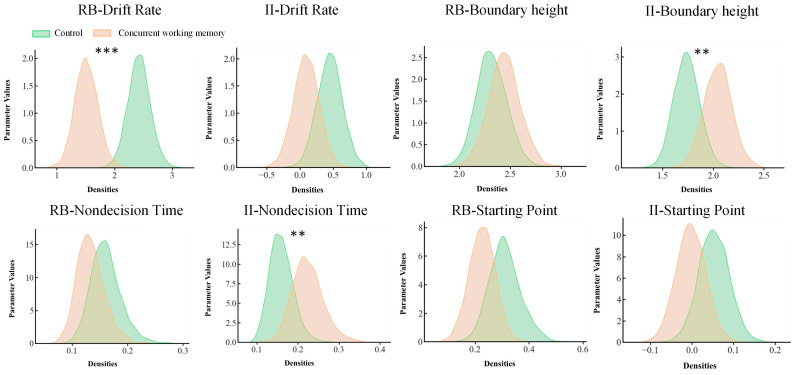
HDDM results for the control and concurrent working memory condition under the rule-based task and the information-integration task. Note: *p* < 0.001 ***; *p* < 0.05 **.

A difference test was conducted on the estimated four posterior parameters (see Figure 5). The results indicated that, in the information-integration task, the decision boundary (*p* = 0.05) for the concurrent working memory condition was significantly higher than that of the control condition, while the nondecision time (*p* = 0.03) was significantly shorter. In the rule-based task, the drift rate of the control condition was significantly greater than that of the concurrent working memory condition (*p* < 0.001). No other significant differences were observed between the concurrent working memory condition and the control condition.

Given that only 7 participants employed the information-integration strategy in the task, we conducted further analysis on their model parameters. For the decision threshold and nondecision time—two parameters showing significant differences between the control and experimental groups—we performed one-way ANOVA across three conditions: control group–rule-based strategy, control group–information-integration strategy, and experimental group–rule-based strategy. For the nondecision time (*F* (2, 44) = 5.39, *p* = 0.008), post hoc tests revealed no significant difference between the control group using the information-integration strategy and the control group using the rule-based strategy (*p* = 0.99). However, the former exhibited significantly shorter nondecision time compared to the experimental group using the rule-based strategy (*p* = 0.03). Additionally, a significant difference emerged between the two rule-based conditions (control vs. experimental groups, *p* = 0.005). Regarding the decision threshold (*F* (2, 44) = 3.95, *p* = 0.058) post hoc analysis indicated a significant difference between the control group–information integration strategy and the experimental group–rule-based strategy (*p* = 0.024), while no such difference was observed between the two rule-based strategy conditions (*p* = 0.15), or between the control group–information-integration strategy and control group–rule-based strategy (*p* = 0.26).

### 3.6. Results in Working Memory Task

Firstly, we conducted a one-sample *t*-test to examine the performance of concurrent working memory tasks in both the rule-based task (*M* = 0.88, *SD* = 0.14) and the information-integration task (*M* = 0.92, *SD* = 0.05), with an 80% accuracy rate as our primary criterion. The results revealed that the accuracy rate of the concurrent working memory task was significantly exceeded 80%, irrespective of whether it was performed in the rule-based task (*t* (21) = 2.56, *p* = 0.02, *d* = 0.56) or the information-integration task (*t* (21) = 10.65, *p* < 0.001, *d* = 2.32). Those results suggested that participants in either the rule-based task or the information-integration task were performing the concurrent working memory task at all. The task difficulty of the concurrent working memory task was compared between the rule-based task and the information-integration task using an independent samples *t*-test on the accuracy of the concurrent working memory task. The results indicated no significant difference in the accuracy of the concurrent working memory task between the rule-based task and the information-integration task, *t* (42) = 1.44, *p* = 0.16, *d* = 0.22. These findings suggested that both rule-based and information-integration auditory category learning were equally affected by the auditory working memory task.

## 4. Discussion

The present study revealed that the concurrent working memory task had a negative impact on accuracy and response time for both the rule-based and information-integration tasks. The categorization strategies model showed that most participants employed a rule-based strategy in both the rule-based and information-integration tasks, but more participants used an information-integration strategy in the control condition than in the concurrent working memory condition during the information-integration task. Furthermore, there was a significant decrease in drift rate and longer nondecision time for the rule-based strategy under the concurrent working memory condition compared to the control condition. For the information-integration strategy in the control group, compared to the rule-based strategy in the experimental group, the degree of caution in the decision-making of participants was lower. These findings suggest that engagement in a concurrent auditory working memory task impacted categorization performance and strategy selection, as it reduced the speed of information accumulation and increased nondecision time for the rule-based strategy while increasing cautious decision-making for the information-integration strategy.

The present study found that the concurrent working memory task interfered with both the rule-based and information-integration tasks. This phenomenon may stem from most participants utilizing a rule-based strategy in both tasks. The categorization strategies model revealed that most participants employed a rule-based strategy in both the rule-based task and the information-integration task, as previous studies have demonstrated processing advantages of auditory stimuli using a rule-based learning strategy (Maddox et al., 2006; Smith et al., 2014). Consequently, the concurrent working memory task is likely to influence performance in both types of tasks. Specifically, according to the results of the strategy analysis under controlled conditions, 92% of participants employed a single-rule strategy for classification in the rule-based task, whereas only 32% opted for the information-integration strategy in the information-integration task. This suggests that participants may encounter difficulties in applying the information-integration strategy during auditory category learning, as 68% did not use this strategy. Consequently, when faced with an additional working memory task, participants were more susceptible to interference. Indeed, none of the participants utilized the information-integration strategy in the information-integration task when required to complete a concurrent working memory task.

This study demonstrated that the majority of participants relied on the rule-based strategy for classification in both rule-based and information-integration tasks within auditory category learning. One plausible explanation for this finding is that participants may favor rule-based strategies when the separation between contrasting information-integration categories is so small that it becomes difficult for them to utilize optimal strategy. Another possible explanation is that participants tend to prefer the rule-based strategy when processing auditory information, a preference supported by our earlier research. Specifically, our previous studies have shown that when rules are presented auditorily and similarity-based strategies are presented visually, significantly more participants choose auditory rule-based classification over visual similarity-based classification. In contrast, no significant differences were observed when rule-related features were presented visually and similarity-related features auditorily, or when both rule-related and similarity-related features were presented visually (Wu et al., 2018, 2020). Future research could further manipulate the task difficulty of the rule-based and information-integration tasks to examine the proportions of individuals employing the rule-based strategy versus the information-integration strategy in response to stimuli presented in visual versus auditory channels during rule-based and information-integration tasks.

Notably, in the rule-based task, a higher proportion of participants employed the optime strategy (the unidimensional rule) in the control condition compared to the concurrent working memory condition. In the information-integration task, more participants used information-integration strategies under the control condition than in the concurrent condition. These research findings indicate that a high cognitive load can negatively affect individuals’ capacity to learn and apply optimal strategies. For instance, studies have shown that in tasks with less structured categories, such as the 5-4 task, where rules are not easily discernible or examples are scarce, participants tend to use similarity-based classification methods (Ashby & Ell, 2001; Smith & Minda, 1998). On the other hand, in tasks with more structured categories and clearer rules, like correlated-cues tasks, participants are more inclined to adopt rule-based classification (Little et al., 2011; Nosofsky et al., 1994). Moreover, computational modeling research has found that when the number of training trials is limited or the learning rate is slow, models rely on similarity for classification. However, as the number of training trials increases or the learning rate speeds up, models transition to rule-based classification (Verguts & Fias, 2009). To explore how working memory influences the choice of classification strategy, researchers conducted comparisons between adults and children. The findings revealed that a significantly larger proportion of adults utilized rule-based classification compared to similarity-based methods, while no significant difference was observed among children. This discrepancy is attributed to the less developed working memory in children, which limits their ability to effectively apply rules for classification (Rabi et al., 2015; Zeithamova & Maddox, 2006).

Considering that most participants employed a rule-based strategy in both the rule-based task and information-integration task, the present study revealed that concurrent working memory task interfered with rule-based category learning. Moreover, employing HDDM analysis for the rule-based strategy, the drift rate was significantly higher, and the nondecision time was significantly lower in the control condition compared to the concurrent working memory condition. A higher drift rate indicates more rapid accumulation of information; a lower nondecision time suggests faster stimulus processing. For the information-integration strategy, the degree of caution in participants’ decision-making was increased. Previous studies found that working memory is related to the speed and accuracy of category learning (Craig & Lewandowsky, 2012; Lewandowsky et al., 2012; McHaney et al., 2021; Souza et al., 2024), and working memory is associated with higher accuracy as well as faster and slightly more cautious decision-making in non-native speech category learning. The DDM results extended previous studies by showing that concurrent working memory tasks influence the speed of information accumulation and the duration of stimulus encoding during rule-based category learning, whereas they impact the level of decision caution during information-integration category learning. This suggests that participants adopting the information-integration strategy required more accumulated evidence before making decisions, reflecting heightened cognitive caution. In contrast, the rule-based strategy showed less variability in decision thresholds, indicating a more stable evidence accumulation process under rule-based frameworks. When using the rule-based strategy, participants can acquire a clear and explicit rule. Consequently, this process may primarily involve the speed of information accumulation and the duration of stimulus encoding. In contrast, when employing the information-integration strategy, participants are more likely to encounter ambiguous stimuli and uncertain rules. As a result, during decision-making in this process, participants may exhibit greater hesitation and caution. Future studies could further dissect the psychological processes involved in implicit learning or procedural learning.

## 5. Limitations

One limitation of this study is the substantial difference in correct rates between the rule-based task and the information-integration task. However, this discrepancy may not undermine the interpretation of the COVIS theory. In this study, both the more challenging information-integration task and the simpler rule-based task were affected by the concurrent working memory task relative to the control condition. Strategy analysis revealed that a greater number of participants opted for the rule-based strategy rather than the information-integration strategy when performing the information-integration task. This preference might explain why the information-integration task was also significantly impacted by the concurrent working memory task. It appears that using the information-integration strategy in the auditory channel poses greater difficulty for participants. Therefore, future studies should consider manipulating the difficulty levels of the information-integration task to further investigate the influence of the concurrent working memory task on auditory information-integration tasks of varying difficulties.

## 6. Conclusions

The present study revealed that the concurrent auditory working memory task impaired both the accuracy and reaction time in auditory category learning. This phenomenon may be attributed to the tendency of individuals to adopt a rule-based strategy for classification in both auditory rule-based and information-integration tasks. Additionally, the concurrent working memory task influenced the categorization strategy selection. Furthermore, DDM results revealed that these impairments primarily manifested as a decrease in information acquisition rate, more nondecision time for the rule-based strategy, and more cautious decision-making related to the information-integration strategy.

## Figures and Tables

**Table 1:** Category-distribution parameters for the auditory stimuli in the rule-based and information-integration tasks.

Category	*M1*	*M2*	*S1*	*S2*	*r*
Rule-based					
A	200 ms	800 Hz	30	30	0
B	900 ms	800 Hz	30	30	0
Information-integration					
A_1_	100 ms	623 Hz	30	30	0
A_2_	300 ms	829 Hz	30	30	0
A_3_	500 ms	1115 Hz	30	30	0
A_4_	700 ms	1361 Hz	30	30	0
B_1_	200 ms	426 Hz	30	30	0
B_2_	400 ms	692 Hz	30	30	0
B_3_	600 ms	938 Hz	30	30	0
B_4_	800 ms	1180 Hz	30	30	0

Note: *M1* represents the mean on the sound duration dimension; *M2* represents the mean on the audio frequency dimension; *S* represents the standard deviation; *r* represents the correlation coefficient.

**Table 2:** The proportion and accuracy of participants in each condition whose data were most effectively explained by each model in the experiment.

Model	UD		CJ		II		RAN	
	PoP	ACC	PoP	ACC	PoP	ACC	PoP	ACC
RB Control	0.92	0.93	0.08	0.98	-	-	-	-
RB Dual Task	0.59	0.88	0.41	0.80	-	-	-	-
II Control	0.45	0.67	0.23	0.59	0.32	0.60	-	-
II Dual Task	0.68	0.53	0.32	0.53	-	-	-	-

Note: Dashes signify the absence of participants’ data in this model. CJ stands for conjunctive; UD for unidimensional; II for information-integration; RAN for random responding; PoP for the proportion of participants; and ACC for the respective strategy.

## Data Availability

The raw data supporting the conclusions of this article will be made available by the authors upon request.
